# Assessing the population genetic structure and demographic history of *Anopheles gambiae* and *Anopheles arabiensis* at island and mainland sites in Uganda: implications for testing novel malaria vector control approaches

**DOI:** 10.1186/s12936-025-05768-x

**Published:** 2026-01-20

**Authors:** Rita Mwima, Tin-Yu J. Hui, Edward Lukyamuzi, Marilou Bodde, Alex Makunin, Krystal Birungi, Martin Lukindu, Ann Nanteza, Dennis Muhanguzi, Mara Lawniczak, Austin Burt, Jonathan K. Kayondo

**Affiliations:** 1 Department of Entomology, Uganda Virus Research Institute (UVRI), Entebbe, Uganda; 2 Department of Biotechnical and Diagnostic Sciences, College of Veterinary Medicine, Animal Resources and Biosecurity (COVAB), Makerere University, Kampala, Uganda; 3 Silwood Park Campus, Department of Life Sciences, Imperial College London, Ascot, UK; 4 Tree of Life, Wellcome Sanger Institute, Wellcome Genome Campus, Hinxton, Cambridgeshire UK

**Keywords:** Gene drive systems, Insecticide resistance, Field trial sites, Gene flow

## Abstract

**Background:**

Despite substantial investments in malaria control, the disease remains a major burden in sub-Saharan Africa, particularly Uganda. Novel tools such as gene drive systems are being developed to suppress malaria vector populations, but their deployment requires detailed knowledge of mosquito population genetics.

**Methods:**

The genetic structure, diversity, and demographic history of *Anopheles gambiae* and *Anopheles arabiensis* were assessed at six sites in Uganda: three islands in Lake Victoria and three mainland sites. A total of 2918 *An, gambiae* and 173 *An. arabiensis* were genotyped using targeted amplicon sequencing of 62 loci across coding and non-coding regions of the genome.

**Results:**

Population structure analyses revealed clear separation between the two species but little differentiation within each species across sites. Pairwise *F*_*ST*_ values among *An. gambiae* populations were low (0.00054–0.028) but often statistically significant, with mainland populations showing higher connectivity and island populations exhibiting greater isolation. *Anopheles arabiensis* mainland populations showed no statistically significant differentiation, suggesting panmixia. Principal component analysis and Bayesian clustering similarly distinguished species-level structure but no obvious substructure within sites.

Mainland *An. gambiae* populations displayed higher nucleotide diversity than island populations, while *An. arabiensis* showed the lowest diversity overall. Tajima’s D values were negative across sites, consistent with recent population expansions. Effective population size estimates indicated small populations at the islands (146–249) compared to large mainland populations (4054–8190).

**Discussion:**

These findings demonstrate strong genetic differentiation between *An. gambiae* and *An. arabiensis*, and subtle but meaningful structure between island and mainland *An. gambiae* populations. The reduced diversity and small effective population sizes at island sites suggest stronger genetic drift and limited gene flow, in contrast to the highly connected mainland populations.

**Conclusion:**

This study highlights how geographic and ecological factors shape mosquito population structure and provides critical evidence for the design and monitoring of genetic-based vector control interventions, including the planning and evaluation of field trials.

**Supplementary Information:**

The online version contains supplementary material available at 10.1186/s12936-025-05768-x.

## Background

Despite substantial investment in malaria control, the disease remains a global burden, especially in the sub-Saharan Africa. This is because of the adaptive dynamics of the *Anopheles gambiae* species complex, together with other factors that include insecticide resistance, drug resistance, limited access to healthcare, and environmental and socio-economic challenges [1, 2].

In 2024, the World Health Organization (WHO) reported about 200 million cases and 600,000 deaths [3]. Recent years have shown a persistently high malaria burden with case and mortality numbers remaining largely stable from 2021 to 2023, underscoring the health challenge the disease poses, despite the control efforts in place [4, 5]. Sub-Saharan Africa carries the greatest burden globally, accounting for 94% of all malaria cases and 96% of all deaths [3]. Uganda, in particular, experiences a heavy burden due to malaria, ranking third in the number of malaria cases worldwide in 2023 and contributing 5% of the global burden [3].

The fight against malaria is increasingly challenged by the evolving resistance of parasites to antimalarial drugs and of mosquitoes to various insecticides, coupled with the partial efficacy of current control methods [3, 6–9]. To achieve malaria elimination and eradication will thus require new products and interventions to be used in tandem with the existing tools [10]. One promising strategy is the mosquito gene drive technology, a genetic engineering approach that biases the inheritance of a specific gene or trait, causing it to spread rapidly through a population over generations, capable of spreading into an entire population from low initial frequencies [11, 12] to either suppress mosquito populations by reducing their fertility or modify them so they are unable to transmit the malaria parasite [13].

Despite the proof-of-concept mosquito transformation studies done in laboratories and the idententification of candidate gene drive systems [16–18], there is need to advance to further field trials such that product feasibility and efficacy can be evaluated. Furthermore, detailed population genetic assessments must be done systematically before deploying any proposed trial or intervention [19]. Information on population genetic structure and gene flow within a vector species provides a basis for modeling the impact of trial intervention, aides in trial design, and helps select suitable locations for field trials [20, 21].

In Uganda, the geographic and genetic isolation of lacustrine *An. gambiae* island populations from mainland renders them suitable candidate sites for initial testing of mosquito gene drive systems [14]. Conversely, regions with high gene flow are significant for gene drive strategies because they provide high connectivity among populations and enhance the potential for rapid and extensive spread of gene drive elements, which is essential for effective malaria vector control on a broader scale [22–24].

Knowledge of *An. gambiae* and *Anopheles arabiensis* (also a member of the *An. gambiae* species complex) genetic diversity, structure, and population sizes at island and mainland sites will provide critical insights into the genetic differences and dynamics at the target field sites, thereby aiding in the design of interventions and monitoring their efficacy [20, 25, 26]. Several studies have examined the structure of *An. gambiae* at the island and mainland sites in Uganda [26, 27, 31], but there are no studies that compare the population structure of both *An. gambiae* and *An. arabiensis*, despite their known sympatric occurrence in many regions [28, 29]. These two species are closely related members of the *An. gambiae* complex, sharing overlapping ranges and habitats, which creates opportunities for gene flow and introgression that could impact important traits such as insecticide resistance and vector competence [20, 30]. Therefore, assessing their population structure and potential gene flow jointly, is critical to understanding their ecological interactions and for optimizing vector control strategies. *Anopheles arabiensis* from the lacustrine islands is often excluded from further analysis due to their limited numbers in the mosquito collections done to date [25, 26, 31], and yet anecdotal observations suggest that the islands on Lake Victoria may have an increasing presence of *An. arabiensis* alongside *An. gambiae*, suggesting a shift in species composition and complexity of malaria vector species in these areas (M. Lukindu, pers. commun.).

This study employed population genetics approaches including measures of genetic diversity, differentiation and gene flow estimates to characterize the population structure of *An. gambiae* and *An. arabiensis* across selected island and mainland sites in Uganda. This analysis utilised data generated from an amplicon panel specifically designed for species identity.

## Methods

### Mosquito sampling

**Fig. 1:**
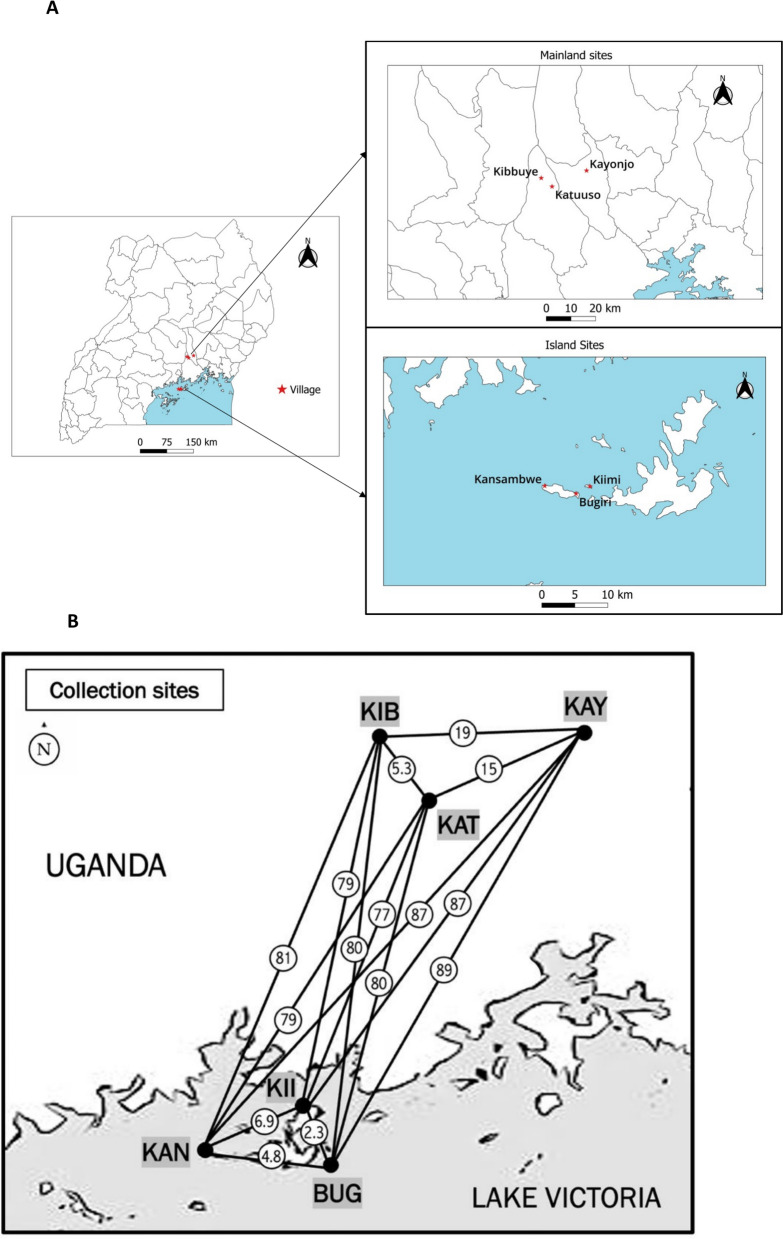
**A** Map showing the location of the 3 mainland and 3 island study sites in Uganda, **B** Distances (in kilometers and circled) between sites. *KAY* Kayonjo, *KIB* Kibbuye, *KAT* Katuuso, *KII* Kiimi, *KAN *Kansambwe, *BUG* Bugiri

Mosquito collections were conducted from six sites including three mainland sites (Kayonjo, Katuuso, Kibbuye) and three island (Bugiri, Kiimi, Kansambwe) sites (Fig. 1). These sites were chosen based on several criteria: (1) high malaria endemicity, (2) a high density of the target mosquito species, (3) a distance from urban areas with a mix of permanent and temporary housing, (4) a moderate population size (between 500 and 1000 residents), (5) minimal ongoing malaria vector control activities, and (6) a cooperative and welcoming local community. Kibbuye and Katuuso villages are situated in Mukono district and Kayonjo village in Kayunga district (Fig. 1), which are each approximately 50 km North West of the capital city, Kampala. The lacustrine island sites are situated within Lake Victoria in Uganda and are connected by ship and ferry service to the Ugandan mainland.

Sampling on the islands and mainlands were part of routine mosquito collections done by the Department of Entomology at the Uganda Virus Research Institute. On the islands, collections were done from April 2013 to April 2016, and on the mainland from January 2016 to December 2018. Adult mosquitoes were sampled indoors and outdoors using human landing catches (HLC), pyrethroid spray catches (PSC), indoor and outdoor aspirators, and catch basin traps (CBT). All mosquitoes were morphologically identified using the *Anopheles* morphological identification keys and then stored in 80% ethanol for subsequent molecular analysis.

### Genomic DNA extraction

A total of 3515 whole mosquito samples, each stored in a 96-well plate of 80% ethanol, were shipped to the Wellcome Sanger Institute, United Kingdom, for DNA extraction and amplicon sequencing [15, 32]. In brief, DNA extraction was completed by removing the ethanol from each specimen, adding 100 ul of lysis buffer C to each, and incubating plates at 56 ℃ for overnight, allowing preservation of specimens for further examination [15]. DNA from each mosquito was then subjected to a single polymerase chain reaction (PCR) containing a 64 primer pair plex [15]. Each reaction then went into a second PCR to add indexing primers. Eight plates of mosquitoes were pooled for a single MiSeq library [15].

Using the ANOpheles SPecies and Plasmodium (ANOSPP) panel of 64 phylogenetically informative and highly variable “amplicon loci” of which 62 target the *Anopheles* nuclear genome and the other 2 targeting the *Plasmodium* mitochondria (note: these amplicon loci are of ~ 160 base pairs or “sites” long in downstream analyses), the generated sequence data enabled species identification, detection of *Plasmodium*, hybrid or contaminated samples, and identification of cryptic species [15]. The panel consisted of 62 nuclear loci distributed across the *Anopheles* genome, comprising 17 coding (exonic), 22 intronic, and 23 intergenic (non-coding) regions [15]. (For all other details regarding the sequencing techniques, and the functions of the 62 amplicon loci, please refer to the original publication [15, 32]).

Species identification was done via a Variational Autoencoder (VAE), which is a machine learning approach that identifies patterns in high-dimensional data by compressing it into a lower-dimensional representation [33]. The VAE was thus used to distinguish between closely related species [32].

### Bioinformatics processing

The raw reads from the generated sequence data for the confirmed *An. gambiae* and *An*. *arabiensis* were processed using AmpSeeker, an open-source computational pipeline which includes quality filtering, primer trimming, alignment to the reference genome file (VectorBase-59_AgambiaePEST_Genome.fasta), and variant calling (e.g. with BWA, bcftools) [33]. AmpSeeker is a Snakemake-based workflow designed for Illumina amplicon sequencing data [34]. Using this pipeline, samples were filtered based on sequencing depth, heterozygosity and principal component outlier analysis to remove low-quality or anomalous samples, before downstream analyses like population structure and gene flow were performed [34].

**Fig. 2:**
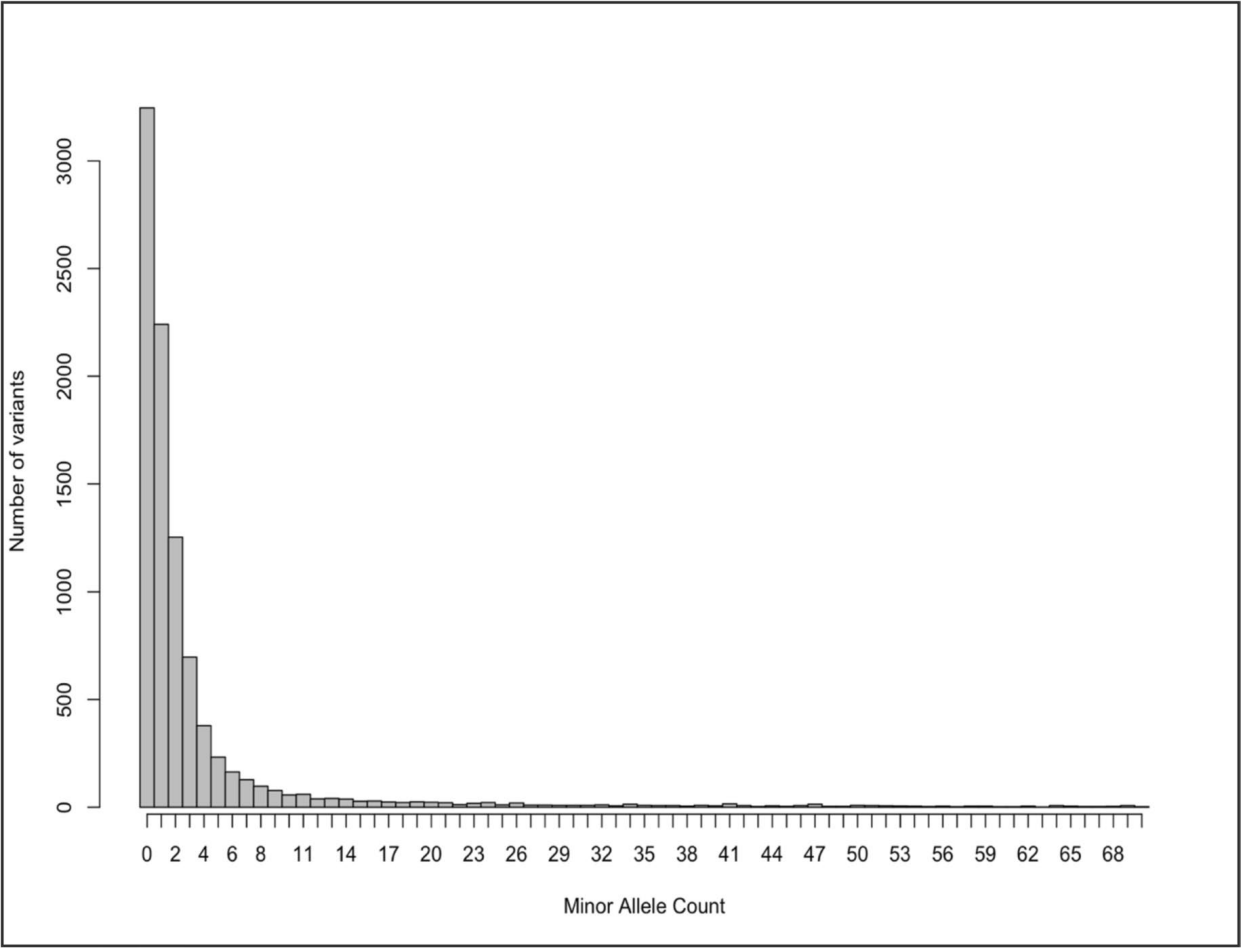
Minor allele frequency (MAF) spectrum between 0 and 2% (for the whole dataset)

**Fig. 3:**
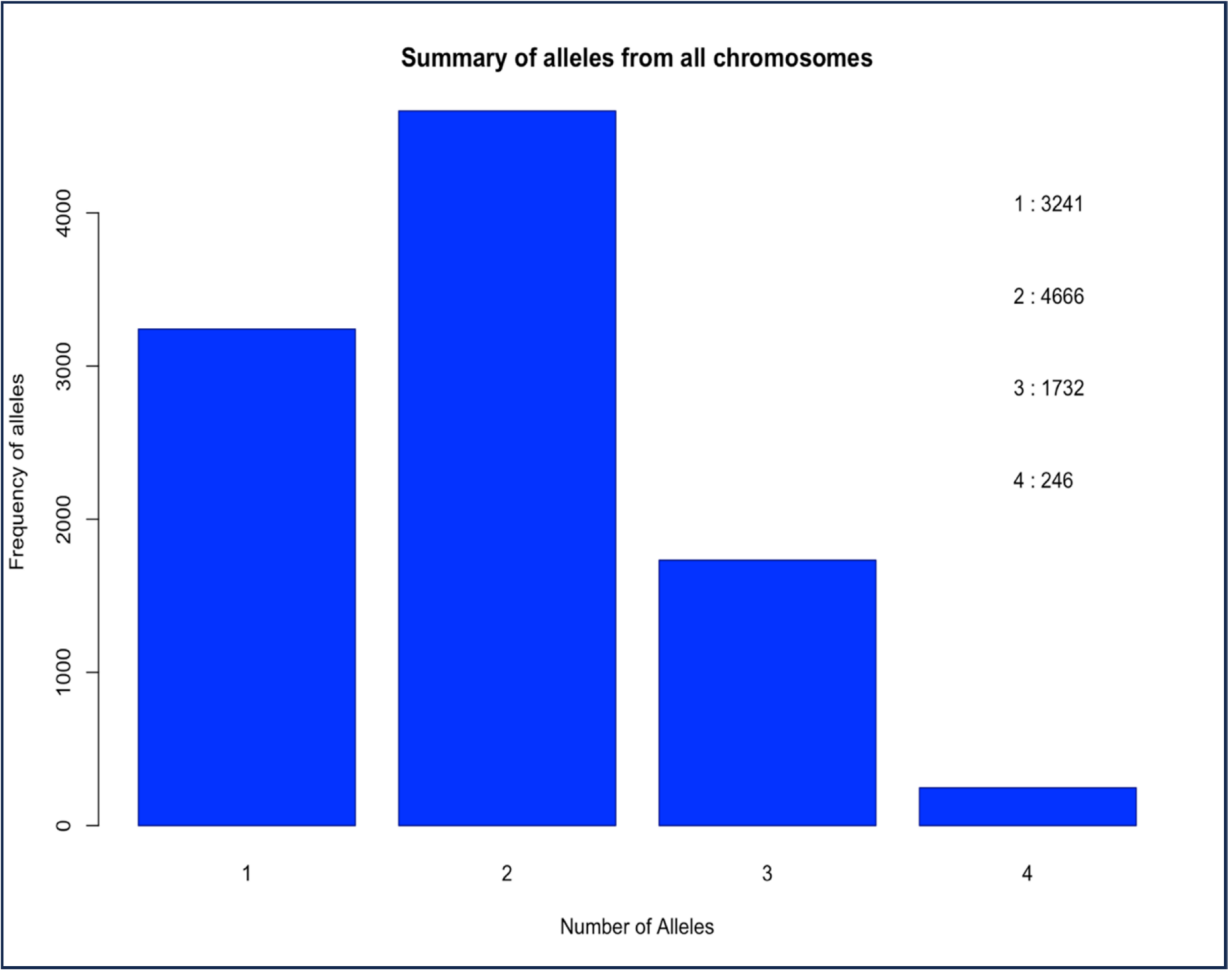
Summary of the number of alleles per site from all chromosomes. 9890 sites are included. The 5 sites with 0 alleles are missing values in all *An. gambiae* and *An. arabiensis* samples and were excluded from the graph

The resulting raw variant call format (VCF) file contained variant calls across all samples and comprises a total of 12,412 sites (inclusive of insertions and deletions (indels), primer regions, fixed and polymorphic sites). Subsequent subsetting was performed on the VCF file based on the metadata information, which provided details about the mosquito samples, including individual sample IDs, species, collection location, collection period, and season among others. In the filtering process, indels were removed, resulting in 9,890 remaining sites. These sites were distributed across the genome as follows: X (n = 952), 2L (n = 1881), 2R (n = 2815), 3L (n = 1615), 3R (n = 2627). The minor allele frequency (maf) spectrum between 0 and 2% is shown in Fig. 2, and the number of alleles per locus (from fixed to quadriallelic) is in Fig. 3. Downstream analyses were based on these 9890 sites with potential further filtering, such as according to maf and missing values (Using only biallelic SNPs).

### Population genomic analysis

#### Population structure

The following methods were used to describe population structure: (1) *F*_*ST*_ between and within pairs of populations using version 1.3.23 of the Popkin package in R [35, 36], (2) Principal Components Analysis (PCA) using version 3.6.2 of the prcomp package in R [35, 36], and (3) Bayesian clustering analysis with STRUCTURE v2.3.4 [37]. Average pairwise Hudson’s *F*_*ST*_ was calculated using the fst_hudson_pairwise() function of the Popkinsuppl package [38], after which standard errors were computed using the leave-one-out approach that is a specific form of the jackknife resampling method [39–41]. Z-scores were then computed by dividing the estimated *F*_*ST*_ by its standard error, then converted into p-values for hypothesis testing [41]. All the *An. gambiae* collected from the same location were considered as a population, while all 173 *An. arabiensis* were grouped as the seventh population because their sizes per site were too small to conduct statistically robust population-level analyses individually. Grouping all *An. arabiensis* samples increased the analytical power to detect genetic patterns and structure across the mainland populations, providing a more reliable overview of their genetic differentiation.

For pairwise *F*_*ST*_, biallelic sites with 10% missing data from within the 3L chromosome with maf >  = 1% were used, while for PCA, all chromosomes were considered and thereafter only the 3L arm, because, unlike other autosomes, it does not carry chromosomal inversions that suppress recombination and could confound analyses of population structure. To investigate the role of geographic distance in shaping genetic differentiation, a Mantel test was used to test for isolation by distance (IBD) between populations by combining Euclidean geographic distances (calculated from geographic coordinates) and genetic distances based on 9999 permutations using GenAlEx 6.51b2 [42, 43]. For *An. arabiensis* populations, the same *F*_*ST*_ and Mantel tests were also conducted using the 3L chromosome arm biallelic SNPs but only for the three mainland sites. The islands were left out because of their small sample sizes (Table 1). PCA was performed in R v4.3.1 with the prcomp() function [44, 45], first for biallelic SNPs from all chromosomes and then for the 3L chromosome arm.

**Table 1:** The number of confirmed *An. gambiae* and *An. arabiensis* samples across six collection sites

	Bugiri†	Kiimi†	Kansambwe†	Kayonjo	Katuuso	Kibbuye	Total
*An. gambiae*	28	258	294	1178	450	710	2918
*An. arabiensis*	2	0	3	51	39	78	173
Total	30	258	297	1229	489	788	3091

^†^ Denotes island sites

Doubletons, defined as sites with exactly two copies of the minor allele in a biallelic site, were analyzed. For these, a contingency table was constructed from observed counts of heterozygous doubleton pairs across populations, and expected counts were calculated based on sample size proportions to assess population structure. Populations were grouped into three categories, *arabiensis* (*An. arabiensis*)*,* mainland *An. gambiae,* and island *An. gambiae*, and a chi-square test was conducted to compare observed and expected counts to detect significant population structure.

To find the optimal number of clusters (K) in STRUCTURE (using model with no admixture), 20 independent runs for each K (from 2 to 8) were conducted, using a burn-in value of 100,000 iterations followed by 100,000 repetitions [37]. Then K was determined using the Delta K method of Evanno et al*.* [46], following which CLUMPAK was used to construct a graphical representation of the genetic structure of the 3091 (both *An. gambiae* and *An. arabiensis*) mosquito samples [47]. In this analysis, one variable site per amplicon locus from the 2R, 3R, and 3L chromosome arms were randomly chosen to minimise genetic linkage, with the constraint that maf ≥ 1%.

### ***Genetic diversity and N***_***e***_

To assess the genetic diversity per population, the overall nucleotide diversity (π) as well as individually for the 6 *An. gambiae* and 3 mainland *An. arabiensis* populations were computed for each chromosome arm [48] using VCF files without missing data. Deviation from the standard neutral model was tested using Tajima’s D, which was performed for each site and chromosome to examine population expansion [49]. Tajima’s D and nucleotide diversity were computed using the Pegas package in R [50]. Estimates of contemporary *N*_*e*_ were attained using the linkage disequilibrium (LD) based method LDNe [51] of NeEstimator v.2.01 [52]. One site per amplicon locus from chromosomes 3R and 3L with maf ≥ 1% were randomly chosen to avoid tight linkage. Nucleotide diversity, Tajima’s D and contemporary *N*_*e*_ were similarly computed for individual mainland *An. arabiensis* populations.

## Results

### Genetic differentiation (*F*_*ST*_)

2918 samples were confirmed as *An. gambiae* and 173 as *An. arabiensis*. These individuals and their resulting amplicon data served as the subjects for downstream population genetic analyses, and Table 1 summarises their distribution across the six collection sites. The average pairwise Hudson’s *F*_*ST*_ is found in Table 2A. All pairwise comparisons produced low *F*_*ST*_ values (0.00054–0.028), except between the “outgroup” *An. arabiensis* and any *An. gambiae* populations. Among *An. gambiae* mainland populations, pairwise *F*_*ST*_ values were very low (0.00054–0.0016, indicating minimal genetic differentiation, and similarly, low *F*_*ST*_ values were observed between Bugiri (island) and the other two islands (Kiimi and Kansambwe), suggesting significant gene flow. The highest *F*_*ST*_ values occurred between Kansambwe (island) and all the mainland sites (Kayonjo, Katuuso and Kibbuye) (island vs mainland), and between Kiimi and Kansambwe (island vs island), implying restricted gene flow in these comparisons (Table 2A). The pairwise *F*_*ST*_ values between Bugiri (island) and all the other sites, Kayonjo (mainland) and Katuuso (mainland), Kiimi (island) and Katuuso(mainland) and Kiimi (island) and Kibbuye (mainland) were low and non-significant (Z < 1.96, p > 0.05). Pairwise *F*_*ST*_ values among the mainland *An. arabiensis* populations (Table 2B) were computed, and low and statistically nonsignificant pairwise *F*_*ST*_ values (−0.0014994 to 0.00785541) were mostly recorded. The *F*_*ST*_ value between Katuuso (mainland) and Kibbuye (mainland) was negative, which is interpreted as no differentiation.

**Table 2:** Pairwise *F*_*ST*_ statistics

*A. An. gambiae* & *An. arabiensis*							
	BUGIRI^†^	KIIMI ^†^	KANSAMBWE^†^	KAYONJO	KATUUSO	KIBBUYE	An. arabiensis
BUGIRI^†^	0	0.680	0.591	1.76	1.63	1.48	4.36*
KIIMI^†^	0.00287	0	4.63*	1.98*	1.94	1.78	4.36*
KANSAMBWE^†^	0.00272	0.0212	0	3.19*	3.04*	3.92*	4.04*
KAYONJO	0.00995	0.0120	0.0272	0	1.20	1.99*	3.78*
KATUUSO	0.0120	0.0148	0.0284	0.00054	0	2.44*	3.83*
KIBBUYE	0.0103	0.0174	0.0243	0.00109	0.00163	0	3.74*
*An. arabiensis*	0.217	0.202	0.263	0.227	0.233	0.209	0

*B. An. arabiensis*			
	KAYONJO	KATUUSO	KIBBUYE
KAYONJO	0	1.82	0.0936
KATUUSO	0.00786	0	−1.071
KIBBUYE	0.000181	−0.0014994	0

(A) Pairwise *F*_*ST*_ from the 3L chromosome arm for the six *An. gambiae* populations, with the 173 *An. arabiensis* grouped as the seventh population. (B) Pairwise *F*_*ST*_ among the three *An. arabiensis* populations. For both tables, the lower left triangle in each table shows average Hudson’s *F*_*ST*_ values between each population pair, while the upper right triangle shows the Z-scores for each *F*_*ST*_ value calculated via a block-jackknife method. The values with * indicate significant *F*_*ST*_ values (Z > 1.96, p < 0.05). † Denotes island sites

**Fig. 4:**
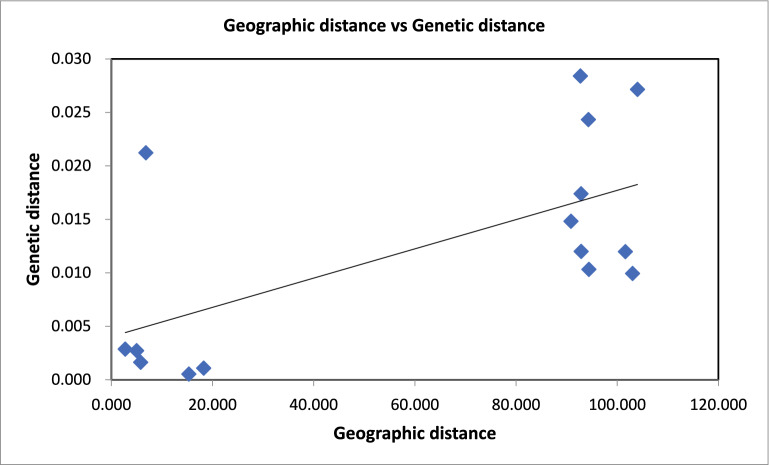
*F*_*ST*_ versus geographical distance (km) for *An. gambiae*. *F*_*ST*_ values were extracted from Table 2. The figure shows a positive slope with no significant correlation between the genetic and geographic distances (p = 0.052)

The pairwise *F*_*ST*_ values for *An. gambiae* were plotted against geographic distances to investigate the strength of isolation by distance [43, 53], and the correlation was not statistically significant (Fig. 4, (p = 0.052).

### PCA

**Fig. 5:**
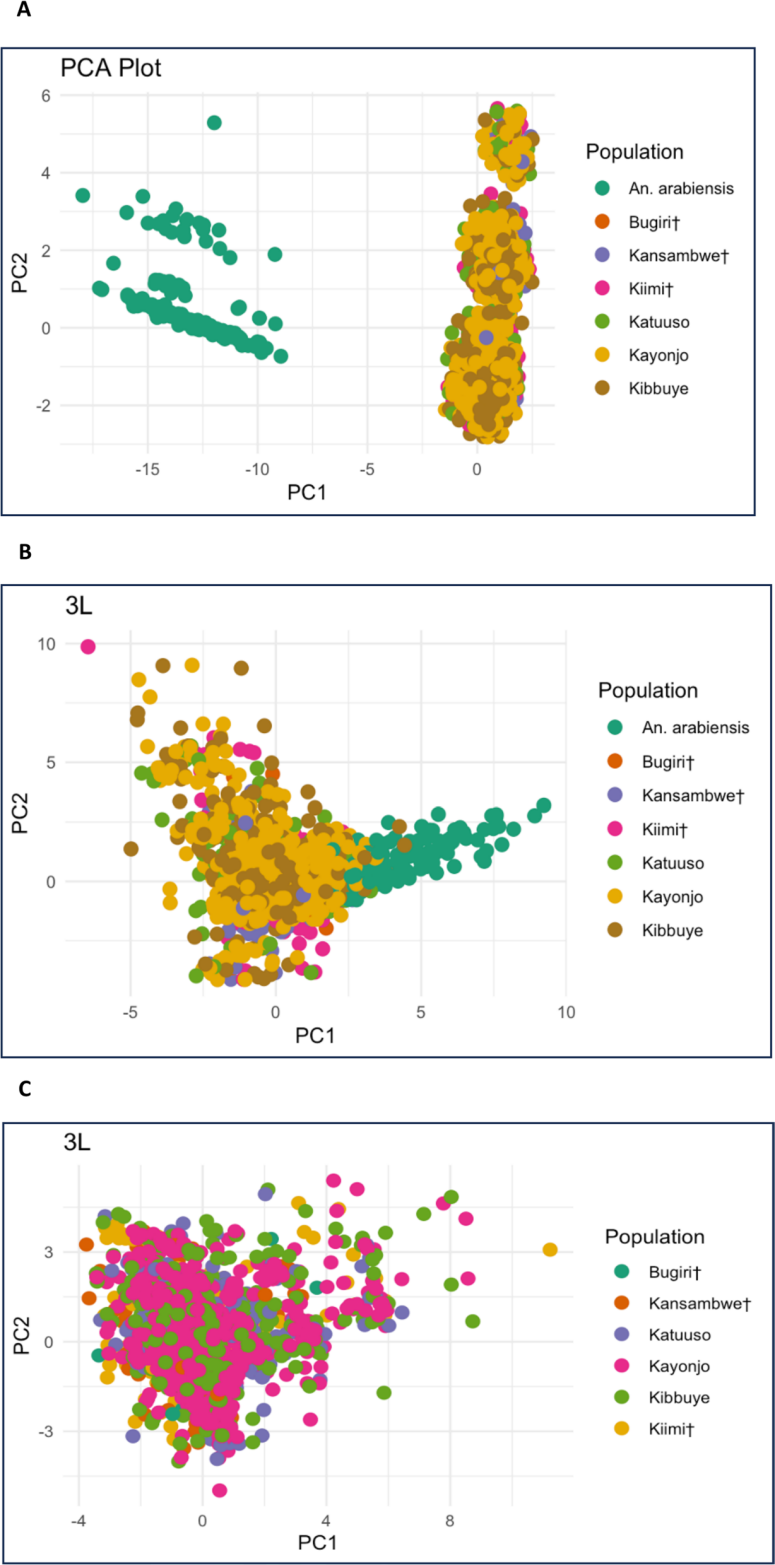
**A** Principal component analysis of all chromosome arms of *An. gambiae* (island and mainland sites) and *An. arabiensis*, **B** The PCA was calculated from the 3L chromosome arm of *An. gambiae* and *An. arabiensis* individuals, and **C**: The PCA was calculated from the 3L chromosome arm of only *An. gambiae* individuals. ^†^Denotes island sites

PCA visualises how the individuals were genetically clustered within and between collection sites. Analysis using all biallelic sites of all chromosome arms of all *An. gambiae* and *An. arabiensis* samples showed clustering into two distinct groups in accordance with each specie’s genetic difference (Fig. 5A). The 3L chromosome arm from both *An. gambiae* and *An. arabiensis* also showed species-specific clustering (Fig. 5B) and using chromosome 3L sites of only *An. gambiae*, no geographical clustering of individuals was observed (Fig. 5C).

### Bayesian clustering analysis

**Fig. 6:**
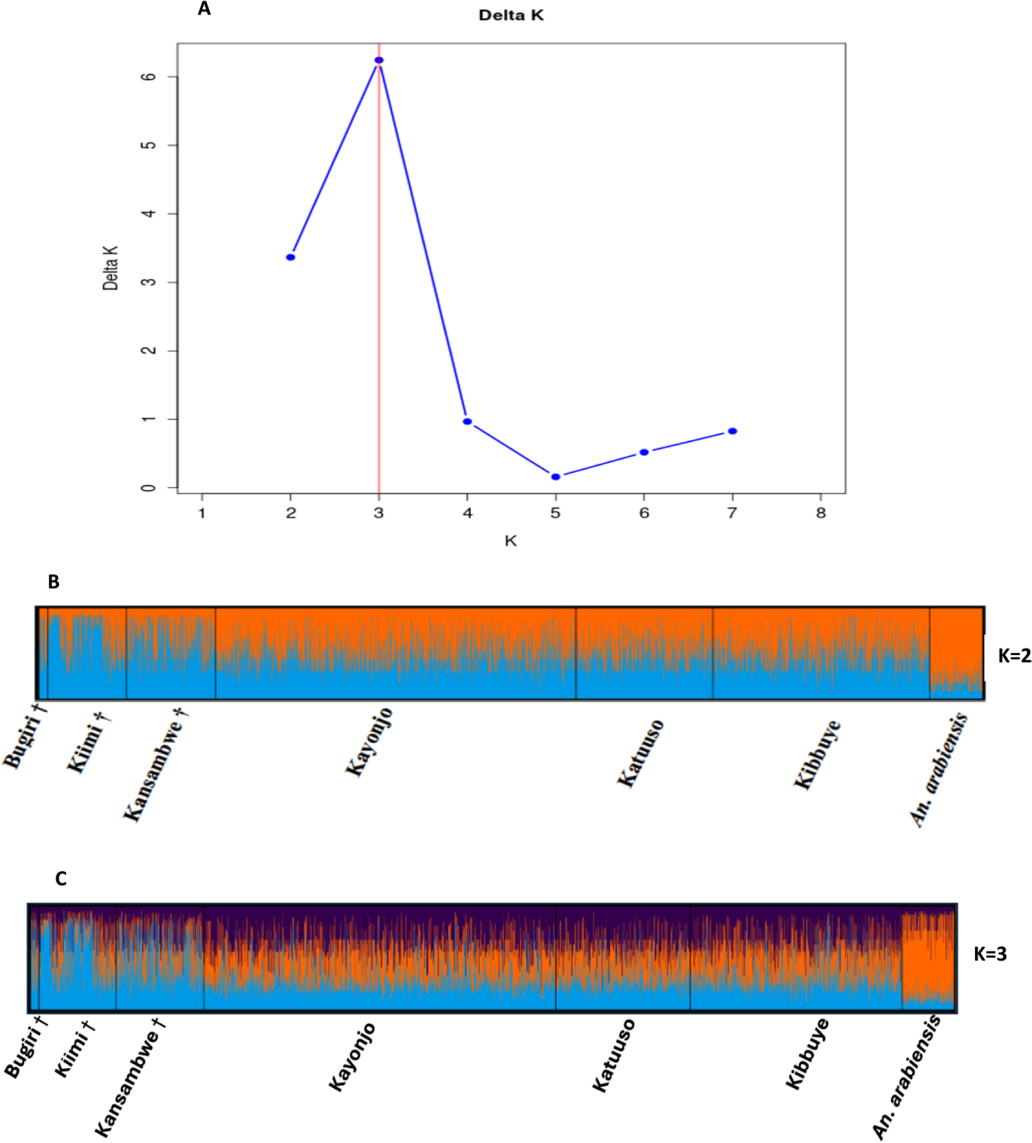
Outputs from Bayesian clustering analysis for the 2918 *An. gambiae* and 173 *An. arabiensis*. **A** The optimal number of clusters (*K* = *3*) based on the Evanno et al. [46] method showing deltaK **B** Bar plot representing K = 2 of 3091 mosquitoes, each represented by a thin vertical bar colored in proportion to their estimated ancestry within each cluster **C** Bar plot representing K = 3 of 3091 mosquitoes. A vertical black line bounds each site and the labels with † for islands while those without are for mainland samples

Based on the log-likelihood values and the DeltaK plot, Κ = 3 was the optimal number of evolutionary clusters (Fig. 6A). Therefore, the six *An. gambiae* populations and pooled *An. arabiensis* were substantially admixed at Κ = 3 (Κ = 3 best explains the genetic variance present between all the sites). Upon close inspection of the results from the Bayesian cluster analysis(Fig. 6B), three significant genetic clusters were identified: (1) pooled *An. arabiensis* individuals, (2) island *An. gambiae* individuals, (3) mainland *An. gambiae* individuals. *An. arabiensis* were assigned to the orange population with the highest probabilities, separating them from all *An. gambiae* mosquitoes. The island *An. gambiae* were mostly assigned to the blue but with slightly more uncertainly. Much of the variation was recorded between populations (*An. gambiae* island and mainland individuals and *An. arabiensis* individuals) instead of among individuals within a site. These results from STRUCTURE (Fig. 6B) were also largely consistent with those from PCA (Fig. 5A–C) with clear separation at an interspecific level and no clear separation at the intraspecific level.

### Allele sharing in doubleton variants

839 pairs of doubletons were identified. The expected counts under the null hypothesis of no population structure (i.e. the expected courts are proportional to sample sizes) were computed. Because of the limited resolution available to distinguish between individual populations in the dataset, the original 6 + 1 (Bugiri, Kiimi, Kansambwe, Kayonjo, Katuuso, Kibbuye, and *An. arabiensis*) population groups were reduced to three broader categories: *An. arabiensis*, mainland *An. gambiae,* and island *An. gambiae*. In this way, more robust statistical comparisons and interpretations of allele sharing across major population segments could be made. The reduction from 6 + 1 populations to three groups improved statistical power and provided insights into gene flow dynamics across the major geographic and species categories of interest.

There were significant deviations between observed and expected pairwise counts of doubleton pairs in the three groups ($\chi^2$=368, df = 4, p = 0.000). The observed pairwise counts of heterozygotes within and between population groups highlight actual genetic similarities, while the expected pairwise counts reflect the hypothetical distribution of pairings in the absence of genetic structuring.

When within-group genetic structuring (Table 3) was considered, the mainland *An. gambiae* group showed a significantly higher within-group count of heterozygous pairs than expected (682 observed vs. 480 expected), which suggests a higher-than-anticipated allele sharing or pairing frequency within this group. The island *An. gambiae* group showed a lower-than-expected level of allele sharing (2 observed vs. 29.54 expected), suggesting limited gene flow with other populations, while, the *An. arabiensis* group showed moderated within-group genetic isolation (15 observed versus 2.63 expected counts), suggesting that it is a relatively closed genetic pool with reduced gene flow to other populations.

**Table 3:** Allele sharing in doubleton variants

A. Observed counts			
	*An. arabiensis*	Island *An. gambiae*	Mainland *An. gambiae*
*An. arabiensis*	15	4	102
Island *An. gambiae*	NA	2	34
Mainland *An. gambiae*	NA	NA	682

B. Expected counts			
	*An. arabiensis*	Island *An. gambiae*	Mainland *An. gambiae*
*An. arabiensis*	2.63	17.62	71.04
Island *An. gambiae*	NA	29.54	238.16
Mainland *An. gambiae*	NA	NA	480.01

Within and between group counts

When between-group genetic structuring was considered, the observed gene flow between *An. arabiensis* and mainland *An. gambiae* groups (102 observed versus 71.04 expected counts) indicated occasional genetic exchange, which could be as a result of ancestral similarities. Island *An. gambiae* versus mainland *An. gambiae* had significantly fewer pairs than expected (34 pairs versus an expectation of 238.16 pairs), suggesting restricted gene flow between the two geographic sites. The *An. arabiensis* versus island *An. gambiae* groups had significantly fewer pairs than expected (4 pairs versus an expectation of 17.62 pairs), suggesting restricted gene flow between the two groups.

### Genetic diversity and neutrality tests

**Fig. 7:**
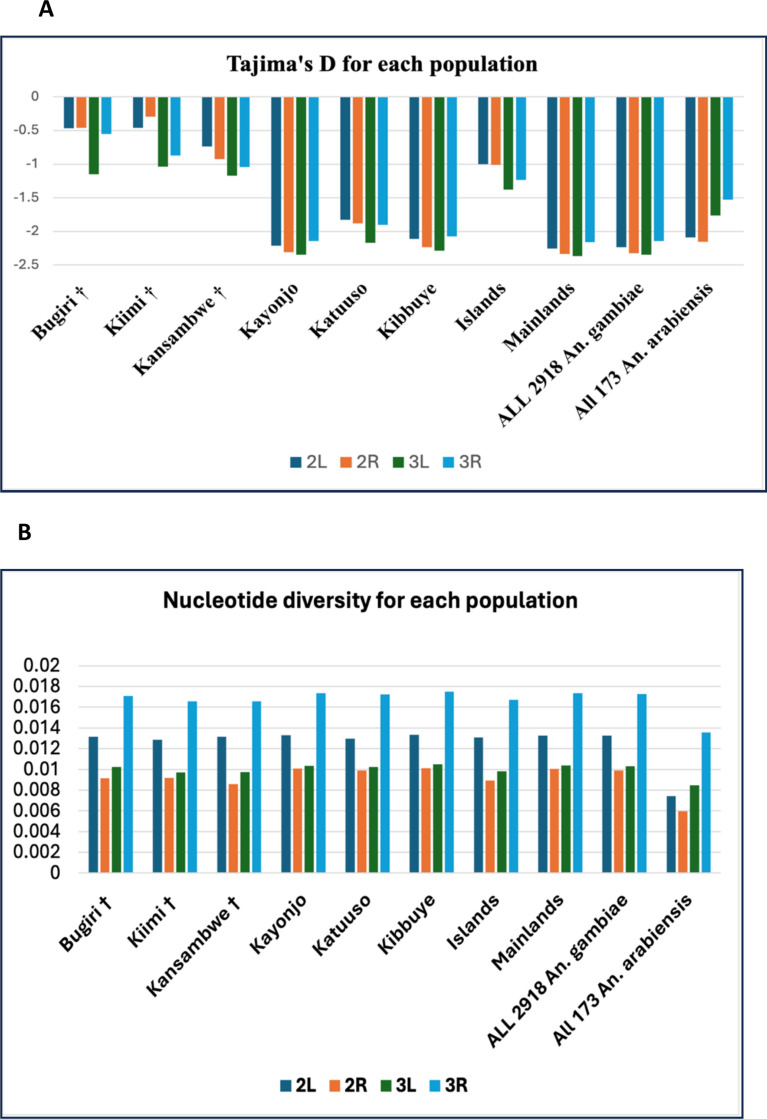
**A** Tajima’s D for the six *An. gambiae* populations per chromosome arm. In addition, the same statistic for the following subsets was computed: pooled *An. gambiae* from the island and the mainland sites, then all *An. gambiae*, and all *An. arabiensis* **B** Nucleotide diversity (π) for the six *An. gambiae* populations per chromosome arm. ^†^Denotes an island site

Nucleotide diversity (pi) and Tajima’s D were calculated for each *An. gambiae* population (SI Table S1), then further for each chromosome arm (Fig. 7A). The overall nucleotide diversity for *An. gambiae* was 0.0115 (range from 0.0107 to 0.01169, SI Table S1) and that of *An. arabiensis* was 0.00859 (ranging from 0.00829 to 0.00859; SI Table 2). Higher diversity values were reported for mainland *An. gambiae* populations (0.01143 to 0.01169; SI Table 1) compared to those for island sites (0.01071 to 0.01103; SI Table 1). The diversity values for *An. arabiensis* mainland populations were lower compared to the mainland *An. gambiae* populations. The pattern of nucleotide diversity compared between chromosomes for different sites (Fig. 7B) was consistent across all sites and chromosome arms (mainland site’s π > island site’s π). At each site, mean chromosomal nucleotide diversity was highest on the 3R chromosome and lowest on the 2R arm. However, pooled *An. arabiensis* (from all locations) had an overall low diversity compared to the different *An. gambiae* populations, with the lowest diversity in the 2R chromosome arm (p = 0.031, Fig. 7B). Overall, the pooled island and mainland diversity falls between the values for each sample site across chromosome arms (Fig. 7B).

Mean Tajima’s D was negative for all sites, an indication of an excess of rare alleles and thus a deviation from the neutral model of a well-mixed population of constant size. However, unlike the *An. gambiae* mainland populations, Tajima’s D values for *An. gambiae* island sites and all *An. arabiensis* populations were not statistically significant from from zero (SI Table S1, S2). When tested for each chromosome and site, all Tajima’s D values were negative. The same was done for the *An. arabiensis* mainland populations (SI Table S2). The Tajima’s D value of the pooled *An. arabiensis* chromosome 2R arm was significantly more negative than other arms of the same site (p = 0.031), and the 2R arm of the individual and pooled populations.

### Contemporary effective population size (*N*_*e*_)

Generally, for *An. gambiae* populations, smaller *N*_*e*_ values were observed for the island sites (between 146 and 249), compared to mainland sites (between 4054 and 8190) (Table 4A). The point estimate for Bugiri was inconclusive but came with a very small lower bound. For the *An. arabiensis* populations, *N*_*e*_ values were estimated only for the mainland sites, and their estimates were comparable to the mainland *An. gambiae* populations. (Table 4B).

**Table 4:** Contemporary *N*_*e*_ estimates

*A. An. gambiae*				
Site	No. of individuals	Number of sites	Overall r^2^	Estimated *N*_*e*_
Bugiri†	28	37	0.035383 $\infty$	$\infty$ (15.1 – $\infty$)
Kiimi†	258	32	0.006168	146.4 (61.7–640.2)
Kansambwe†	294	32	0.004764	249.3 (146.7–538.5)
Kayonjo	1178	15	0.000901	6723.1 (515.9—$\infty$)
Katuuso	450	27	0.002181	4054.1 (640.9—$\infty$)
Kibbuye	710	21	0.001416	(521.5—$\infty$)

*B. An. arabiensis*				
Site	No. of individuals	Number of sites	Overall r^2^	Estimated *N*_*e*_
Kayonjo	51	31	0.018910	$\infty$ (49.4–$\infty$)
Katuuso	39	35	0.027836	3402.8 (36.3—$\infty$)
Kibbuye	78	31	0.013438	3587.4 (95.9–$\infty$)

Site choice is described in the main text. ^†^Denotes island sites. The lowest allele frequency used was 0.01. The values in parentheses represent the jackknife range

## Discussion

### Polymorphic sites and population structure

From the 62 amplicon loci, 9890 sites were mapped onto the reference genome, many of which were fixed sites (Fig. 3) or weakly polymorphic due to the conserved nature of the primer-binding regions (Fig. 2). Despite this limitation, the data revealed useful insights into the population structure of *An. gambiae* and *An. arabiensis* in Uganda. Overall, genetic differentiation among the *An. gambiae* populations was generally low regardless of the sites (*F*_*ST*_ < 0.05), though statistically significant in some cases, suggesting subtle structure with ongoing connectivity.

The high gene flow observed between Bugiri (island) and other sites could be influenced by several factors. One potential cause is human-mediated transport, as frequent boat traffic and ferries between Bugiri and other sites can inadvertently facilitate mosquito dispersal [26, 54, 55]. Additionally, prevailing seasonal wind and weather patterns might assist mosquito flight over water, enhancing connectivity [56–58]. These mechanisms together contribute to the low genetic differentiation reported for Bugiri compared to other island populations. However, the low *F*_*ST*_ value observed for Bugiri could also be influenced by its relatively small sample size, which can increase sampling variance and reduce the power to detect subtle genetic differentiation [58, 59].

In contrast, *An. arabiensis* mainland populations exhibited very low and often negative *F*_*ST*_ values, which is interpreted as *F*_*ST*_ = 0, consistent with unrestricted gene flow [39, 60, 61]. The negative *F*_*ST*_ values (between Katuuso and Kibbuye) likely reflects greater within-site genetic diversity than between-sites genetic diversity, indicative of stable and large *N*_*e*_ [62, 63].

One notable feature of the results is the presence of low but statistically significant *F*_*ST*_ values among many population pairs, indicating that while the genetic differentiation is subtle, structure nonetheless exists. *F*_*ST*_ values for island *An. gambiae* species were however higher compared to mainland sites, reflecting restricted gene flow across water barriers, smaller population sizes, and potential local adaptation comparable to what was reported in previous studies [25, 26, 31]. This could be justified by the large mainland population, given that *F*_*ST*_ is approximately 1/(1 + 4*N*_*e*_*m*) at equilibrium, where $m$ the migration rate [63]. Therefore, applying the large estimated *N*_*e*_ values from mainland populations, the low *F*_*ST*_ values between sites indicate substantial gene flow (migrants per generation), reinforcing the genetic connectivity among them [59, 63]. Given that these *F*_*ST*_ values are significantly greater than zero demonstrates that the populations are not panmictic or identical but exhibit genuine population structure [64, 65]. Recognizing this subtle but meaningful structure is crucial when considering the design and spread dynamics of gene drive or insecticide resistance alleles.

Looking at this from a malaria vector control perspective, high gene flow is a double edged sword. On one hand, it promotes the spread of gene drive constructs throughout connected mosquito populations, potentially enabling rapid spread of desired genetic traits [66]. Conversely, it complicates containment, given that, genetic modifications could unintentionally spread beyond target areas, raising ecological and regulatory concerns. Moreover, convergent evolution may promote the rise of resistance alleles that impair gene drive function, necessitating careful monitoring [67].

It is thus important to distinguish between whether low differentiation results are from ongoing gene flow, convergent adaptation, or both, and understanding these dynamics allows for better-informed strategies to optimize gene drive deployment, balancing efficacy with biosafety in malaria vector control.

Low point estimates of pairwise *F*_*ST*_ yielded high Z-scores, suggesting the presence of subtle yet statistically significant population structure despite low apparent differentiation. This occurrence can largely be attributed to the statistical power afforded by a large sample size, which can detect even minor genetic differences across sites [68, 69]. Between Kiimi and Katuuso, the low pairwise *F*_*ST*_ value and non-significant p-value could be because of temporal sampling variability, which could result in capturing different allele frequencies due to temporal fluctuations [43, 70, 71]. These comparisons of the relative amounts of gene flow taking place between sites, aid in predicting the trajectory of the alleles introduced by gene drive into the wild-type population [20].

The Mantel test results ( p= 0.052) suggested only weak evidence of isolation by distance [72], suggesting that geographic distance partly influences gene flow, which could thus be factored into spatially targeted vector control interventions [59]. Therefore, according to the IBD hypothesis, there may be a meaningful association between the genetic and geographical distances among populations. Therefore, tailored intervention deployment considering spatial genetic structure can optimise control effectiveness.

PCA distinguished species but did not reveal clear geographical structure within *An. gambiae*. These findings align with doubleton analysis (Table 3), which highlighted relative isolation of island sites and stronger connectivity at mainland sites.

The moderate within-group isolation of the *Arabiensis* Group (Table 3) indicates more frequent genetic interactions within this group than expected, potentially due to localized genetic structures, ecological factors, or other selective pressures driving allele frequencies. This could reflect a localized population structure which may arise from ecological factors like microhabitat variation, behavioural differences or localized selective pressures, that can restrict gene flow at finer spatial scales despite the specie’s wide distribution.

The counts between *An. arabiensis* and mainland *An. gambiae* groups indicate a degree of gene flow, implying occasional genetic exchange, which could have direct implications for vector control, especially given the differential patterns of gene flow and inbreeding detected across these sites. This pattern of observed versus expected doubleton pairs sides with the broader view of population structure in malaria vectors, where low gene flow across ecological boundaries frequently results in discrete genetic clusters [73], and has implications on both gene drive strategies and management of insecticide resistance.

STRUCTURE analysis identified three major clusters (Fig. 6) corresponding to species and geography, consistent with *F*_*ST*_ and PCA results. STRUCTURE results reflect what is often observed when there is low genetic differentiation [74]. STRUCTURE output for the *An. gambiae* and *An. arabiensis* species at each site showed sharing of ancestry between each individual at differing proportions (with differing consistency between all island, mainland *An. gambiae* and *An. arabiensis* individuals). The three genetic clusters corresponded to first species (*An. arabiensis*) then to geographical (mainland and island *An. gambiae*) differences, with higher similarity between island compared to mainland *An. gambiae* individuals (Fig. 6B). These results are comparable to the *F*_*ST*_ and PCA results that show less divergence between island and mainland *An. gambiae* individuals and more diversity (clear separation) between *An. gambiae* and *An. arabiensis* individuals [48, 49].

Despite using very few polymorphic sites, STRUCTURE was able to outline the three clusters with visible differences. Therefore, using more sites could produce clearer and more distinct clustering. And because pairwise *F*_*ST*_ is sensitive to allele frequency differences between sites and works best when populations are distinct and genetically isolated, it may not always capture subtle population differentiation, while STRUCTURE, on the other hand, assigns individuals to clusters based on their multilocus genotypes, and is thus more sensitive to subtle population differentiation [37, 64, 75, 76].

### Genetic diversity and neutrality tests

Negative Tajima’s D values (SI Tables S1&S2), especially at mainland sites, suggested an excess of low frequency alleles, potentially reflecting recent population expansion or purifying selection [30, 49, 77]. These results were in line with previous findings in datasets from Uganda and beyond [73]. Given that *An*. *gambiae* thrives in human-altered landscapes, its population and range will thus expand most significantly in peri-urban zones and rural areas with high population density whether on island or mainland sites [78].

The observed differences in Tajima’s D and nucleotide diversity between island and mainland *An. gambiae* populations provide further evidence that these are distinct populations rather than a single panmictic unit. Mainland populations exhibited significantly higher nucleotide diversity and more negative Tajima’s D values, consistent with recent population expansion and larger effective population sizes [63, 79]. In contrast, island populations showed marginally lower nucleotide diversity and Tajima’s D values not significantly different from zero (SI Table S1, S2), suggesting small but stable effective population sizes or mutation-drift equilibrium, with no population bottleneck or expansion, an occurrence consistent with the neutral mutation hypothesis [80, 81]. These genetic differences, together with the subtle but significant population structure detected by *F*_*ST*_ and clustering analyses, support the conclusion that island and mainland *An. gambiae* populations are genetically differentiated and should be considered as separate population units for vector control considerations [26, 31].

These estimates were within the range of values reported in previous studies which utilised four loci (white, tox, G6pd, xdh) across several African locations [82] and immune-related (LRIM1, CTL4, CTLMA2, APL2) and housekeeping genes [83], and were consistent with the fact that populations at island sites display marginally lower nucleotide diversity compared to the mainland [84, 85]. The explanation for this could be inbreeding, or higher degree of isolation with small neighbourhood size [84, 86–88].

On the 2R chromosome arm, the pooled *An. arabiensis* population has lower nucleotide diversity than individual or pooled *An. gambiae* populations, which could be because the 2R arm has many segregating inversions, which can limit recombination in certain regions, leading to reduced genetic diversity over time [89, 90]. This phenomenon is especially pronounced in species like *An. arabiensis*, where these inversions (2La, 2Rb and 3Ra) could be fixed, and further decrease genetic variation along the 2R chromosome relative to *An. gambiae* populations [91, 92].

Additionally, the frequencies for inversions segregating in both species could be/are different for the two species [90], suggesting a decline in population size and/or balancing selection, a feature of the maintenance of segregating 2Rb, c, d, u and j inversions [78, 93].

### Contemporary effective population size

*N*_*e*_ estimates were smaller for island *An. gambiae* than at mainlands sites, reflecting isolation and stronger drift, results comparable to those reported in the studies by Kayondo et al*.* and Wiltshire et al*.* [26, 27, 31]. The low *N*_*e*_ estimates at island sites could be because of their small neighbourhood size and are suggestive of higher levels of genetic differentiation (presented as pairwise *F*_*ST*_ estimates) shown in Table 4A [52].

Mosquito populations at these smaller, more isolated sites are more vulnerable to genetic drift, but may also be more amenable to localized control interventions.

The infinity values of point estimates such as those recorded for Bugiri, was likely due to insufficient sample size compared to the magnitude of genetic drift. Some *N*_*e*_ estimates from mainland sites had infinite upper confidence bounds, which statistically allows the rejection of a ‘no genetic drift’ scenario [94–96].

Larger *N*_*e*_ in the mainland *An. gambiae* populations suggest higher resilience to vector control interventions and more likely to sustain the spread of resistance alleles [97–101]. This implies slower genetic drift which allows for sustained genetic variation that enhances adaptation to environmental changes, and because dry season surviving populations can rebound and maintain genetic diversity across seasons, this further complicates efforts to control malaria vector populations [102–106].

### Implications for malaria vector control

These findings have important implications for: (1) insecticide resistance: high connectivity among mosquitoes at mainland sites facilitate rapid spread of resistance alleles, where as island isolation may slow spread and allow for localised resistance management, (2) Gene drive strategies: the reduced *N*_*e*_ and relative isolation of mosquitoes at island sites lower the release threshold for gene drives, increasing the likelihood of successful establishment [20]. Relative isolation and smaller *N*_*e*_ values favours islands as contained experimental sites for gene drive trials, allowing more manageable monitoring and containment [66]. Conversely, large, well-connected mainland sites would require larger releases and careful management to ensure effective drive propagation, and (3) Vector elimination efforts: The weak but detectable structure among *An. gambiae* populations suggests that control efforts need to account for ongoing gene flow between sites. Local elimination on islands may be feasible, but reinvasion from the mainland remains a threat.

The ease of access to the islands, higher genetic differentiation, genetic structure, smaller effective population sizes compared to mainland sites, and their small geographical size, makes islands promising sites for field trials to test the effectiveness of mosquito gene drive systems.

*Anopheles arabiensis* individuals are often excluded from further analysis due to their limited numbers in the mosquito collections done to date [25, 26, 31], which could be because of the sampling methods used. Therefore, studies that target the exophilic and zoophagic characteristics of this species should be carried out to give a better picture of its density, specifically at the islands which are the potential sites for field trials to test mosquito gene drive systems. This will help us to confirm the proportion of the non-target malaria vector species at the island sites.

Future work should utilize whole-genome sequencing data and imcoporate temporal sampling. Further investigations should also clarify the specific contributions of the factors examined here, as well as the influence of other biotic factors (e.g., passive transport, chromosome inversions and seasonality) and abiotic factors that may contribute to the observed differentiation. These will add a valuable body of information required for evaluating these Islands as potential release sites for mosquito gene drive systems.

## Conclusion

Despite limited polymorphism in the loci studied, this work highlights subtle but meaningful population structure between *An. gambiae* and *An. arabiensis* populations. The contrast between small, isolated island sites and large, connected mainland sites has direct consequences for the design of insecticide resistance management strategies, feasibility of gene drive interventions, and broader malaria vector control approaches.

## Supplementary Information

Supplementary material 1

## Data Availability

The data set analysed in this paper is available on Github: https://github.com/tusubirarita/Genetic-diversity-Population-structure
