# Supplementary material for: Assessing the population genetic structure and demographic history of Anopheles gambiae and Anopheles arabiensis at island and mainland sites in Uganda: implications for testing novel malaria vector control approaches
Source: Malar J. 2026 Jan 20;25:96. doi: 10.1186/s12936-025-05768-x (PMC12903332; doi:10.1186/s12936-025-05768-x)
Supplement: Supplementary file 1 — Supplementary material 1 [file 12936_2025_5768_MOESM1_ESM.docx]

**Supplementary Information (SI)**

**Table S1.** Nucleotide diversity and Tajima’s D for the six *An. gambiae* populations. The last row is all *An. gambiae* (2918) combined for comparison. Site choice was as described in the main text. The asterisk * denotes statistical significance, (p < 0.05). † Denotes an island population.

| **Population** | **Nucleotide diversity (pi)** | **Tajima’s D** |
| --- | --- | --- |
| Kayonjo | 0.01163 | -2.3069* |
| Katuuso | 0.01143 | -2.0219* |
| Kibbuye | 0.01169 | -2.2356* |
| Kiimi † | 0.01081 | -0.8223 |
| Kansambwe † | 0.01071 | -1.0827 |
| Bugiri † | 0.01103 | -0.6954 |
| All *An. gambiae* (Mainland & Island) | 0.01150 | -2.3073* |

**Table S2.** Nucleotide diversity and Tajima’s D for the three *An. arabiensis* populations

The last row is all *An. arabiensis* (173) combined for comparison. Site choice was as described in the main text.

| **Population** | **Nucleotide diversity (pi)** | **Tajima’s D** |
| --- | --- | --- |
| Kayonjo | 0.00859 | -1.17475 |
| Katuuso | 0.00829 | -0.98993 |
| Kibbuye | 0.00847 | -1.73386 |
| All *An. arabiensis* | 0.00859 | -1.91156 |
